# Can a Chatbot Help Heal a Wound? Context-Aware Prompts for Boosting Adherence in Diabetic Foot Ulcers

**DOI:** 10.3390/diabetology7050096

**Published:** 2026-05-12

**Authors:** Aria Rabet, Aminreza Khandan, Arian Rabet, Mohammad Dehghan Rouzi, Fabiola Rodriguez, Adriana Garibay, David G. Armstrong, Bijan Najafi

**Affiliations:** 1 Center for Advanced Surgical & Interventional Technology (CASIT), Department of Surgery, University of California, Los Angeles, CA 90024, USA; 2 Southwestern Academic Limb Salvage Alliance (SALSA), Department of Surgery, Keck School of Medicine, University of Southern California, Los Angeles, CA 90033, USA

**Keywords:** diabetic foot ulcer, offloading, wearable sensors, remote monitoring, mobile health, context-aware notifications, feasibility

## Abstract

**Background:**

Smart offloading technologies enable the real-time, objective monitoring of adherence in patients with diabetic foot ulcers (DFUs). Although remote tracking may reinforce adherence and improve wound healing, effectiveness depends on sustained device use, particularly as devices are often removed during rest periods. Real-time, behavior-contingent feedback informed by sensor data, including AI-supported messaging capable of detecting nonadherence, may enhance reinforcement. However, the feasibility and behavioral impact of such strategies remain unclear.

**Methods:**

We conducted a prospective feasibility case series nested within a larger DFU cohort of 210 participants, enrolling eight adults with active DFUs. Participants used a sensor-integrated offloading device paired with a smartwatch (SmartBoot) and a mobile application (CORA) that delivered notifications to their smartphones. Notifications were either schedule-based or context-aware, using real-time SmartBoot data to generate personalized messages. The primary outcome was a sensor-detected transition from nonadherent to adherent offloading within 60 min.

**Results:**

A total of 130 notifications were delivered, with 125 included in the behavioral response analysis. Context-aware notifications demonstrated higher transition rates than schedule-based notifications. Adaptive Reinforcement yielded the highest response rate (77.4%, 24/31), followed by Clinical Course Correction (71.4%, 20/28), whereas Safety and Technical Assurance (40.7%, 11/27) and Motivational Coaching (30.8%, 12/39) showed lower response rates.

**Conclusions:**

Real-time, context-aware feedback is feasible and associated with improved short-term adherence, supporting evaluation in larger trials.

## 1. Introduction

Offloading remains fundamental to diabetic foot ulcer (DFU) care, yet consistent adherence to prescribed devices is frequently suboptimal [1–5]. While non-removable modalities (e.g., casts) can improve adherence, their limited comfort and acceptability constrain widespread use [4,6–8]. In response, a new generation of digitally enabled offloading systems has emerged, integrating wearable sensors and connected health platforms into removable boots, shifting management from passive prescription to active engagement [1,4,6,9,10]. Among the most notable examples is the SmartBoot system, introduced in the early 2020s [11,12]. This device combines a conventional removable offloading boot with embedded inertial sensors linked to a Bluetooth-enabled smartwatch or smartphone. The system continuously detects boot wear during weight-bearing activity and monitors metrics such as steps, standing time, and gait cadence [11,12]. Data is transmitted in real time to the user’s device and securely stored in the cloud, enabling both patient-facing feedback and remote clinical monitoring. A distinguishing feature is its closed-loop feedback design. When ambulation occurs without the boot, the system issues immediate alerts, such as vibration or audible prompts, encouraging corrective action. Some implementations incorporate a simple gamified interface: a smiling face appears on the smartwatch when the boot is worn appropriately during activity, switching to a sad face when unprotected walking is detected [9,13]. This visual cue leverages behavioral psychology principles, reinforcing adherence through instant, emotionally intuitive feedback. Rather than relying solely on retrospective counseling during clinic visits, SmartBoot provides continuous, real-time nudges that promote sustained protective behavior.

Despite these advances, important gaps remain. The effectiveness of closed-loop systems still depends on sustained engagement with both the boot and its digital interface [6,8]. Removable devices are frequently taken off during rest, sleep, hygiene, or brief activities, and patients may forget to put them back on before resuming weight-bearing. This vulnerability is amplified when the smartwatch, the primary feedback interface, is also removed, uncharged, or disconnected. In such scenarios, the feedback loop is interrupted precisely when reinforcement is most needed [14]. Technical and educational barriers further limit real-world effectiveness [1]. Patients may encounter challenges such as charging batteries, maintaining Bluetooth connectivity, or re-pairing devices after disconnection. Even minor technical issues can reduce trust and sustained engagement. Beyond technical factors, adherence is shaped by behavioral and psychosocial factors, including device burden, mobility limitations, perceived benefit, treatment fatigue, and daily routines [1,15–18]. Competing daily demands, stress, or treatment fatigue may lead patients to deprioritize device use, while frustration or overwhelm can result in disengagement.

Early clinical studies show chatbot responses can achieve patient satisfaction comparable to physician-delivered information [19–21]. A recent survey of internal medicine physicians found favorable attitudes toward AI alongside low technical knowledge, underscoring the need for evidence-based feasibility studies before clinical deployment [22]. Conversational agents embedded within mobile platforms could provide on-demand education, guide patients through troubleshooting steps, reinforce the importance of offloading, and deliver motivational support tailored to individual behavior patterns [21]. However, generic or context-blind reminders risk being perceived as intrusive or impersonal [19,23–25]. This is particularly relevant in DFU care, where adherence barriers are multidimensional [18,26]. Objective monitoring studies further show that protective-device adherence is context-dependent, with use varying by location and time of weight-bearing activity [2,18,27]. Without situational awareness and empathetic framing, automated prompts may fail to resonate with patients’ lived experiences and may not be intuitively accepted [19]. Therefore, while AI-enabled support is promising, its effectiveness depends on contextual intelligence, personalization, and integration within a patient-centered reinforcement strategy [28,29]. Importantly, current chatbot-based interventions in chronic disease management have largely focused on education and self-management support, with limited integration of objective behavioral data. To date, no study has evaluated the feasibility and acceptability of a context-aware chatbot informed by continuous, sensor-based monitoring of adherence to offloading in patients with active diabetic foot ulcers. Such an approach has the potential to enable personalized, timely, and behaviorally informed interventions, thereby enhancing adherence and improving clinical outcomes.

Building on gaps in sustained engagement, technical usability, and context-sensitive reinforcement, we conducted a feasibility study to determine whether behavior-responsive prompting could enhance the closed-loop offloading model. Within an ongoing prospective randomized controlled trial (RCT) in DFU evaluating SmartBoot effectiveness (ClinicalTrials.gov ID: NCT04460573), we performed a nested case series study using a sensor-integrated SmartBoot system (Sensoria Health Inc., Redmond, WA, USA) paired with a mobile application (CORA, Thrive360ai, San Francisco, CA, USA [30]). The platform enabled real-time classification of offloading behavior and delivery of either scheduled reminders or context-aware notifications triggered by detected nonadherence. This approach introduces a context-aware, behavior-contingent feedback strategy within a closed-loop framework, moving beyond traditional schedule-based reminder systems. We hypothesized that behavior-contingent prompts would elicit greater short-term corrective responses than fixed, time-based reminders. The primary objective was to evaluate operational feasibility, including sensing fidelity, notification delivery, and synchronization of behavioral and feedback data, to inform future efficacy trials.

## 2. Materials and Methods

### 2.1. SmartBoot Study

This study was conducted as a nested multiple-case feasibility study embedded within a parent multi-site RCT (ClinicalTrials.gov ID: NCT04460573) of DFU patients. The SmartBoot system, described in our preliminary study [12], is an advanced offloading platform designed to support DFU management through real-time monitoring and feedback. It integrates a removable offloading device with the Sensoria Core (Sensoria Health Inc., Redmond, WA, USA), a six-degree-of-freedom inertial measurement unit with Bluetooth Low Energy connectivity. Data are processed locally via a paired Android-based smartwatch customized and programmed by Sensoria Health Inc. (Redmond, WA, USA), functioning as an edge computing hub, enabling on-device detection of offloading adherence and calculation of key metrics such as protected/unprotected steps and cadence. Patients receive immediate visual feedback to reinforce adherence to prescribed offloading, while data are transmitted to a secure cloud dashboard developed by Sensoria Health Inc. for clinician access, supporting remote monitoring and personalized care. The parent RCT enrolled 210 adults receiving offloading therapy for DFUs, while the present analysis was limited to participants included during the pilot implementation phase (March–May 2025), during which context-aware notifications were delivered to the patients.

### 2.2. Participants

Participants included in this feasibility analysis were adults enrolled in the parent study (ClinicalTrials.gov ID: NCT04460573). Eligible participants were adults (≥18 years) recruited from five outpatient clinics in the Los Angeles metropolitan area (CA, USA) who had an active diabetic foot ulcer (DFU) requiring offloading therapy. Additional inclusion criteria included the ability to provide informed consent, receipt of at least one digital notification through the CORA mobile application (Thrive360ai, San Francisco, CA, USA), with synchronized SmartBoot sensor data and notification logs available. Exclusion criteria followed the parent protocol and included ulcer duration greater than one year, hemoglobin A1c greater than 12%, major vascular disease requiring revascularization, ulcers involving exposed bone or tendon, or non-diabetic lower-extremity ulcers. All participants provided written informed consent prior to enrollment. The study was approved by the University of Southern California Institutional Review Board (protocol code HS-20-00526; initially approved 11 August 2020) and conducted in accordance with the Declaration of Helsinki and Good Clinical Practice guidelines. A total of eight participants met the eligibility criteria and were included based on their exposure during the pilot implementation period and the availability of synchronized sensor and notification data (Table 1).

### 2.3. Experimental Protocol

**Figure 1.**
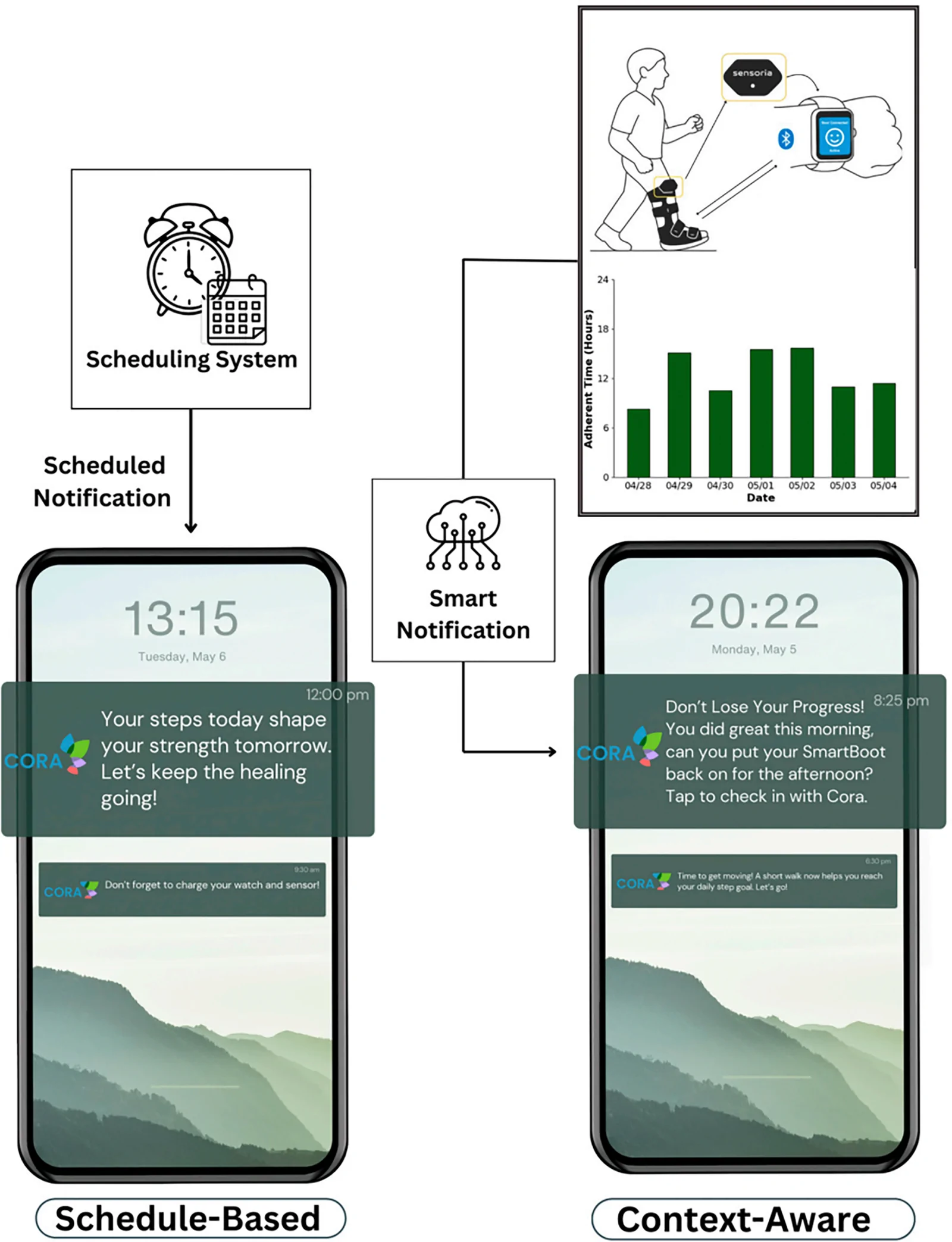
Overview of the SmartBoot–CORA system architecture and feedback loop. The SmartBoot device, equipped with embedded sensors, continuously captures weight-bearing activity and offloading behavior, transmitting data via Bluetooth to a paired smartwatch. Sensor data is processed hourly to inform CORA mobile notifications, which are delivered to the patient’s personal smartphones. Notification type and tone are tailored based on detected adherence patterns and patient status. The system supports both schedule-based notifications, delivered at predefined times, and context-aware notifications, triggered dynamically in response to sensor-confirmed nonadherence.

This study represents a subgroup feasibility analysis derived from a larger parent study (the SmartBoot study described in Section 2.1), aimed at informing the design of future, larger-scale studies. Participants used an instrumented SmartBoot offloading device equipped with embedded Sensoria activity sensors to capture weight-bearing activity and boot-wear status during daily ambulation continuously. Sensor data were transmitted wirelessly via Bluetooth to a paired smartwatch, which functioned as an edge-processing hub and transmitted to a secure cloud infrastructure. The cloud platform supported data storage, retrospective visualization, and clinician-facing summaries. On-device processing included detection of boot wear versus non-wear, classification of ambulatory activity, and computation of step counts. Processed data was used to generate related notifications, which were then transmitted to the patient’s personal smartphone running the CORA mobile application. To contextualize the flow of data from sensing to notification delivery, a schematic overview of the SmartBoot–CORA system architecture and feedback loop is provided below (Figure 1).

### 2.4. Notification Pathways and Categorization

Building on this sensing and data transmission framework, the CORA application implemented two predefined notification pathways designed to deliver notifications to patients under different conditions. Schedule-based notifications were delivered at predetermined times, independent of sensor input. In contrast, context-aware notifications were triggered by SmartBoot system-detected nonadherence, providing activity-contingent feedback aligned with patient behavior. Notification pathways and intent categories were defined to ensure consistent interpretation and analysis (Table 2). Notifications were classified as responsive if a sensor-confirmed transition to adherent behavior (any sensor-detected protected weight-bearing or rest during unprotected ambulation while the watch is active) occurred within 60 min of delivery. The 60 min window was selected to ensure that participants had sufficient time to realistically respond to the notification and initiate the recommended behavior (i.e., resuming offloading) while still capturing short-term, behaviorally meaningful changes under real-world conditions. Notifications without a qualifying behavioral change within this time window were categorized as non-responsive.

### 2.5. Statistical Analysis

All analyses were exploratory and intended to characterize behavioral response patterns rather than to support confirmatory statistical inference. Each notification served as the unit of analysis, with repeated measures clustered within participants. Notification effectiveness, defined as a binary outcome (responsive vs. non-responsive), was analyzed using population-averaged generalized estimating equations (GEE) with a logit link and binomial distribution to account for within-subject correlation [31,32]. GEE models provided population-averaged estimates of notification responsiveness while accounting for correlation among repeated notifications from the same participant [31]. The primary predictor was notification pathway (context-aware vs. schedule-based), with time of day (morning, afternoon, evening) included as a covariate to adjust for daily variation in behavior. An exchangeable correlation structure was specified at the participant level. Odds ratios (ORs) with 95% confidence intervals (CIs) were reported, and time-specific contrasts were derived from the fitted model. For comparisons of response proportions, standardized effect sizes were quantified using Cohen’s h. Given the exploratory design and small sample size, *p*-values were interpreted descriptively, consistent with guidance for pilot and feasibility studies [33]. Additionally, daily adherence time (in minutes) was compared between notification and non-notification days using within-participant Wilcoxon signed-rank tests. Finally, exploratory analyses assessed associations between individual characteristics (e.g., age, education, functional status) and notification responsiveness. All analyses were performed in Python (v3.11; Python Software Foundation, Wilmington, DE, USA) using the statsmodels package (v0.14). Figures were produced with Matplotlib (v3.10).

## 3. Results

All eight participants in this nested feasibility study completed the parent study (100% retention), compared to 120 of 210 participants (57.1%) in the parent randomized controlled trial. Six of eight participants (75%) achieved complete wound healing: two by week 2, one by week 5, one by week 7, one by week 8, and one by week 12. Two participants completed monitoring without achieving full healing during the study period. Adherence time was higher on days when notifications were delivered, with an average within-participant increase of approximately 210 min (3.5 h) compared to days without notifications (Wilcoxon signed-rank test, *p* < 0.05), interpreted descriptively given the exploratory design.

### 3.1. Behavioral Response Analysis

**Figure 2.**
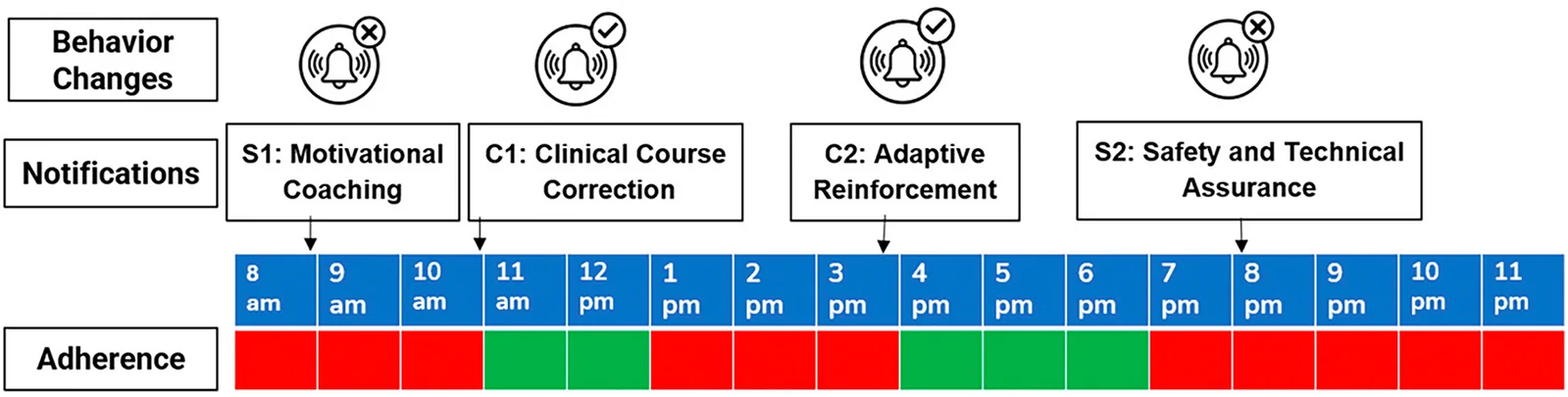
Notification success was assessed based on patient adherence to prescribed offloading following notification delivery. Check (**✓**) and cross (**✗**) icons indicate the presence or absence of the intended behavior change after a given notification. Green blocks represent notifications followed by adherent behavior, whereas red blocks indicate nonadherence. A notification was classified as successful if adherence was observed within a 60 min window after notification delivery.

Behavioral response was assessed using time-stamped SmartBoot sensor data, with adherence defined as either active/rest on boot and rest off boot within 60 min of notification delivery. Figure 2 presents an illustrative example from a single participant, showing notification delivery and corresponding behavioral outcomes across four predefined intent categories: motivational coaching (S1), clinical course correction (C1), adaptive reinforcement (C2), and safety and technical assurance (S2). Green blocks indicate adherence to the therapy, while red blocks indicate non-adherence behavior.

Across eight participants, a total of 130 digital notifications were delivered during the monitoring period. Of these, 125 notifications were eligible for analysis of behavioral response, excluding five schedule-based notifications (three safety/technical assurance and two motivational coaching) that were delivered during periods of baseline adherence. Time-stamped SmartBoot data were available continuously, enabling synchronized evaluation of offloading adherence following notification delivery.

Behavioral response rates varied by notification pathway and intent (Table 3). Context-aware notifications were consistently associated with higher rates of nonadherence-to-adherence transitions compared to schedule-based notifications. Specifically, adaptive reinforcement messages resulted in a 77.4% response rate (24/31), and clinical course correction messages in 71.4% (20/28). In contrast, response rates for schedule-based notifications were 40.7% (11/27) for safety and technical assurance and 30.8% (12/39) for motivational coaching. While context-aware notifications outperformed schedule-based notifications overall, no observable differences in behavioral response rates were observed between subcategories within each pathway (S1 vs. S2 and C1 vs. C2).

**Figure 3.**
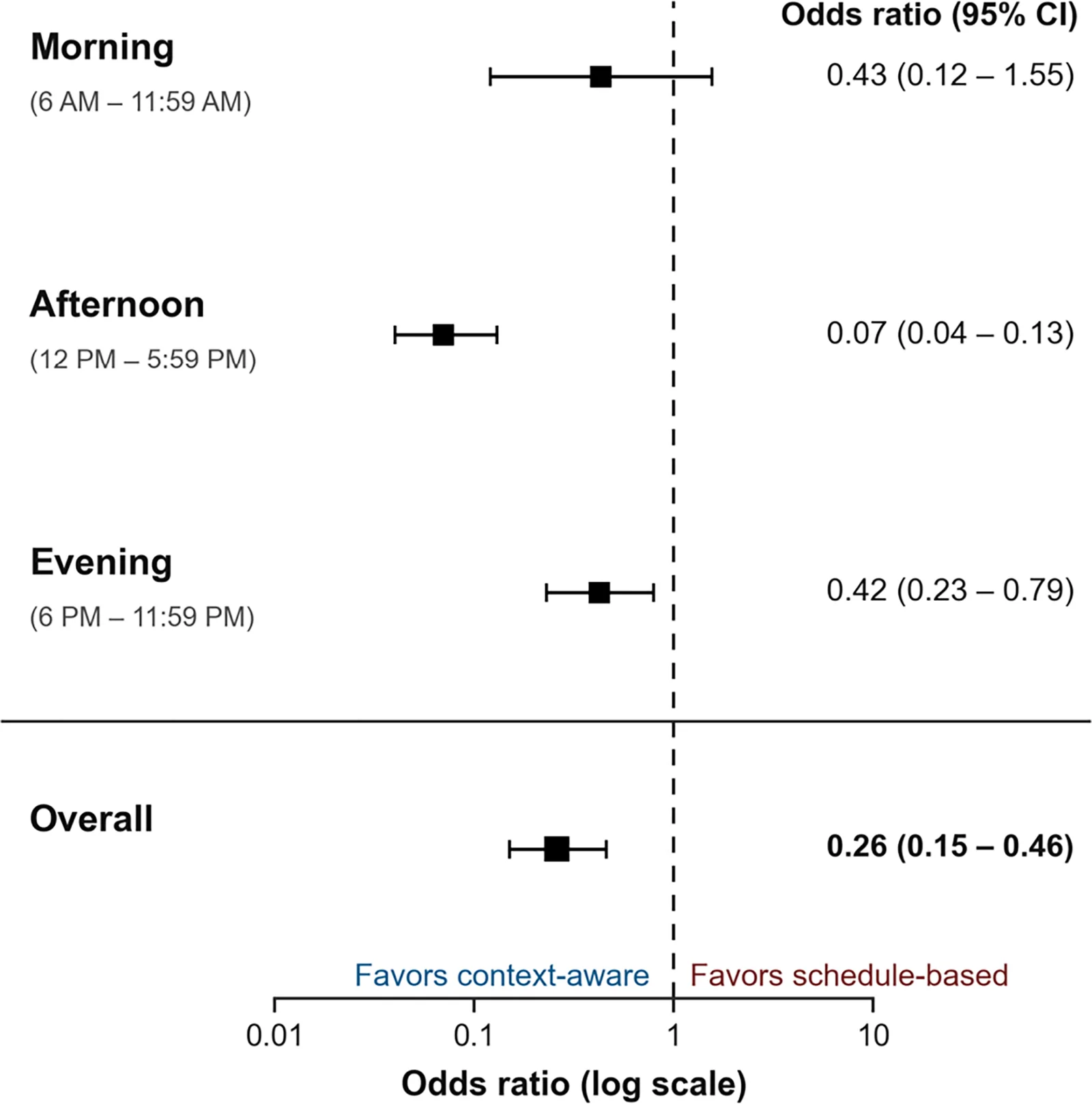
Adjusted odds ratios for behavioral response comparing schedule-based versus context-aware notifications across time-of-day strata. Squares represent the stratum-specific odds ratios for morning, afternoon, and evening, and horizontal lines indicate the corresponding 95% confidence intervals; the diamond represents the overall pooled estimate. The vertical dashed line denotes no difference (OR = 1). Odds ratios < 1 indicate higher effectiveness of context-aware notifications relative to schedule-based delivery.

Furthermore, context-aware notifications were more effective than schedule-based notifications even after adjustment for time of day, with consistent directionality across all time windows. After adjustment for time of day, notification tone showed directional differences in effectiveness, with consistent directional differences observed across time periods. Schedule-based notifications were consistently less effective than context-aware notifications. Specifically, schedule-based notifications showed substantially lower odds of effectiveness compared with context-aware notifications in the afternoon (OR 0.07, 95% CI 0.04–0.13, *p* < 0.01) and in the evening (OR 0.42, 95% CI 0.23–0.79, *p* < 0.01). In the morning, the association was directionally consistent but did not reach statistical significance (OR 0.43, 95% CI 0.12–1.55, *p* = 0.20, Figure 3). Context-aware notifications yielded higher response rates than schedule-based notifications (71–77% vs. 31–41%), corresponding to an absolute increase of approximately 35–45 percentage points. This difference represents a large effect size (Cohen’s h = 0.82).

Finally, exploratory analyses assessed associations between individual characteristics (age, education, functional status) and notification responsiveness. There were no statistically significant main effects for age, college, or independence (*p* > 0.08). Total notifications had a positive (but not significant) association with age, controlling for other variables (*p* = 0.08); due to the limited sample size, these were descriptive and not powered for inference.

### 3.2. Case Summaries

**Figure 4.**
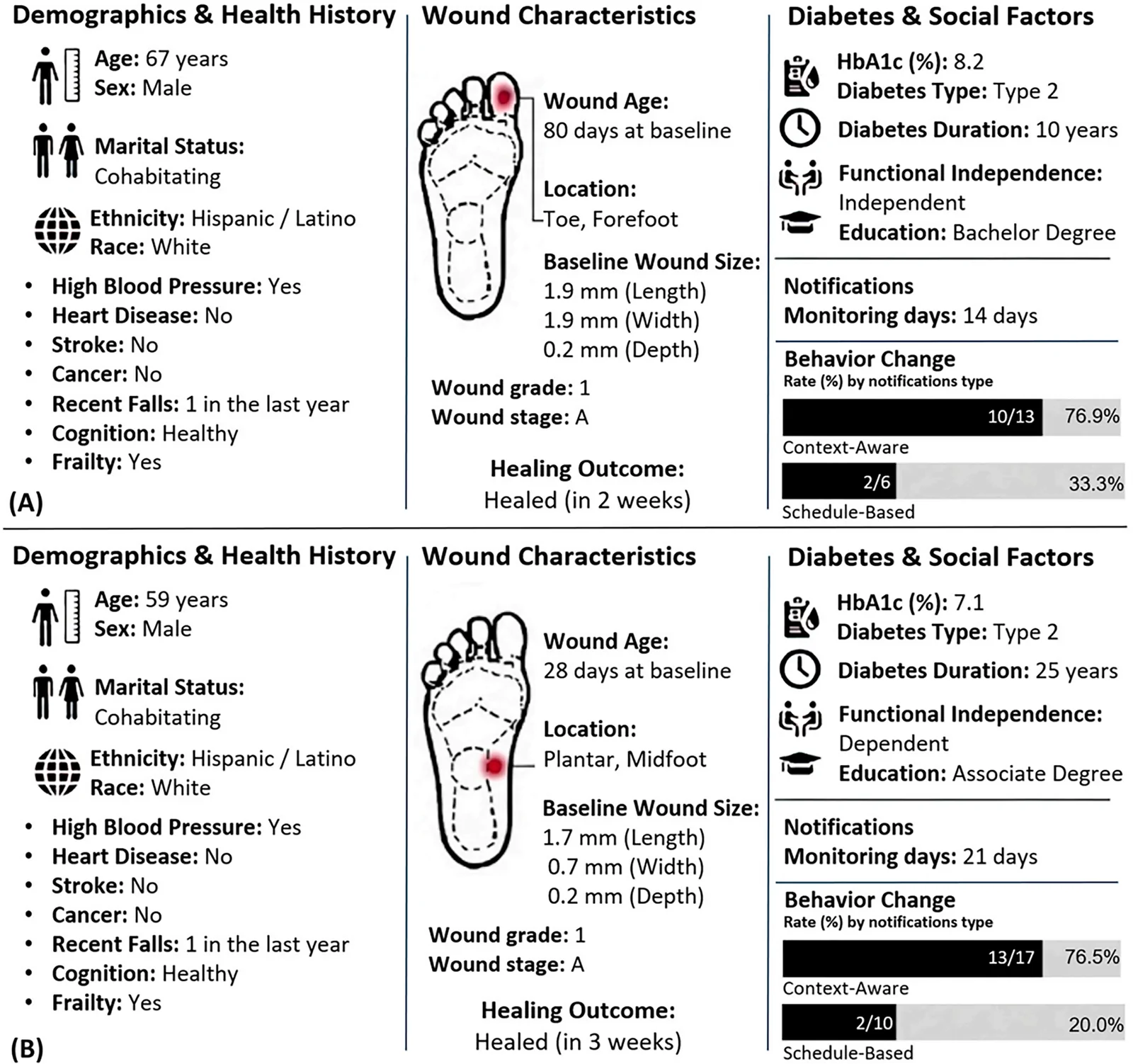
Representative case profiles illustrating notification adherence patterns and wound healing outcomes. Panels (**A**,**B**) present baseline demographic, clinical, and wound characteristics, as well as diabetes-related factors and notification responsiveness for two individuals with diabetic foot ulcers. Adherence is reported as the proportion of context-aware and schedule-based notifications followed by sensor-derived behavior change during the monitoring period. Both cases demonstrated high responsiveness to context-aware notifications and achieved complete wound healing, within two weeks in Panel (**A**) and within three weeks in Panel (**B**).

Case summaries are included to provide high-resolution, patient-level insight into behavioral variability and system interaction patterns, rather than to support broader inference or generalization. To contextualize system performance at the individual level, two representative cases are presented to illustrate behavioral response patterns across notification types. Case 1 was a 67-year-old, functionally independent male with a forefoot ulcer of 80 days’ duration. He primarily used the boot indoors with limited community ambulation. Case 2 was a 59-year-old, functionally dependent male with a midfoot ulcer present for 28 days at baseline. He reported improved mobility while using the boot and thus increased participation in daily activities. Both patients’ ulcers were classified as UT class 1A and achieved wound healing within two to three weeks. Across 14 and 21 monitoring days, respectively, both participants received a combination of context-aware and schedule-based notifications. Behavioral response to context-aware notifications was similar across cases: 76.9% (10/13) in Case 1 and 76.5% (13/17) in Case 2. Scheduled notification adherence was lower in both cases, at 33.3% (2/6) and 20.0% (2/10), respectively. Figure 4A,B illustrate baseline characteristics and their behavioral response patterns.

## 4. Discussion

In this nested feasibility study within a SmartBoot DFU RCT, we evaluated a digital notification system integrated with a mobile app on the patient’s personal smartphone. The system was designed to deliver context-aware and schedule-based notifications and detect short-term changes in the offloading behavior of individuals with DFUs using a SmartBoot system [12]. All eight participants completed the parent study, substantially higher than the completion rate (57.1%) observed in the overall parent cohort. Among 130 notifications, context-aware messages were associated with higher proximal behavior changes than schedule-based prompts, and their effectiveness was consistent across times of day. Additionally, daily adherence time was numerically higher on days when notifications were delivered, with an average increase of 210 min compared to days without notifications (Wilcoxon signed-rank test, *p* < 0.05), interpreted descriptively. While not designed to assess long-term adherence or causal inference, this early-phase evaluation demonstrates the feasibility of detecting and responding to real-time behavioral risk in outpatient DFU care. Consistent adherence to offloading is a cornerstone of DFU management and is strongly emphasized in major clinical guidelines (e.g., ADA Standards of Care [5] and international DFU guidance [34]). In this context, the behavioral responses observed here should be interpreted as proximal indicators of adherence rather than direct measures of clinical effectiveness.

This feasibility analysis also provides high-resolution, time-stamped characterization of behavioral responses to context-aware feedback, supporting the value of reporting these early implementation findings to inform future, adequately powered studies. A major challenge in DFU management is that protective behavior frequently lapses outside clinical supervision [35,36]. Previous digital interventions in DFU care have largely emphasized education and adherence monitoring through patient-reported outcomes or delayed clinical indicators [9,10,37,38]. By integrating real-time sensor monitoring with notification workflows to detect offloading lapses [11,12,39,40], this system directly addresses interruptions in the closed-loop feedback that occur when devices are removed, uncharged, or disconnected. The observed proximal corrective responses suggest that context-aware feedback can provide reinforcement as soon as unprotected weight-bearing occurs, supporting sustained engagement during routine daily activities outside clinical supervision. Rather than assessing wound healing or therapeutic efficacy, this work emphasizes upstream behavioral data, positioning context-aware feedback as a foundational component of adherence support strategies [25,38,39,41]. These findings further support the operational potential of real-time, objective behavioral tracking as a precursor to a trial of technology-assisted outpatient management. From an implementation science perspective, the successful deployment of context-aware digital tools in outpatient DFU care requires attention not only to technical feasibility but also to end-user accessibility, workflow integration, and scalability; factors that should be systematically evaluated in future implementation trials.

Another challenge in DFU care is that sustained adherence is required for healing [42,43]. While longitudinal adherence trajectories were not formally addressed in this feasibility study, the consistent increase in adherence on notification days (an average within-participant increase of 3.5 h) suggests a directional signal toward improved adherence. Clinical outcomes in this small cohort were also encouraging; however, the study was not designed to evaluate treatment effectiveness, and larger controlled studies are needed to determine whether behavior-responsive feedback translates into improved healing. This study did not evaluate sustained adherence or clinical endpoints such as wound healing, time to healing, or recurrence. Behavioral changes observed in this study should be interpreted as indicators of short-term engagement rather than durable adherence or therapeutic success [37,44]. Transitions from nonadherence to adherence within the response window indicate proximal behavioral changes but do not necessarily predict long-term clinical outcomes [45], consistent with the multifactorial nature of DFU recovery [38,46]. Accordingly, these responses may serve as early indicators of patient engagement that could help guide clinical management strategies. Future studies should determine whether repeated proximal responses translate into sustained adherence and improved clinical outcomes in DFU care. Repeated short-term improvements in adherence may contribute to improved wound healing trajectories, although this relationship has not yet been established and requires evaluation in adequately powered studies.

The successful deployment of SmartBoot and CORA demonstrates the feasibility of integrating real-time behavioral monitoring into outpatient DFU management and other clinical settings requiring continuous patient supervision [12,47]. By enabling synchronous sensor and notification pipelines, this platform lays the groundwork for scalable remote adherence support that may be integrated into outpatient DFU workflows [9–11,48]. Real-time data accessibility and interpretability can enhance clinician decision-making and facilitate timely interventions, particularly during periods of risk. Similar approaches may apply to other conditions requiring sustained patient supervision. The core architecture demonstrated here—real-time sensing, context-aware feedback, and behavioral response monitoring—has potential applications beyond DFUs, where real-time engagement with prescribed devices or activities is critical [14,49]. Future research should also explore adaptive feedback strategies that evolve with patient behavior to mitigate habituation and support long-term engagement.

This study has several limitations requiring consideration. First, the analysis was conducted in a small feasibility cohort of eight patients during the pilot implementation phase. The cohort lacked demographic diversity, consisting entirely of male participants, and lacked race and ethnicity diversity, which restricts the ability to evaluate potential effects of sex or race/ethnicity and limits applicability to broader DFU populations. Future studies in larger, more diverse randomized controlled trials are required to evaluate the effectiveness of context-aware notifications. Second, outcomes were assessed at the notification level rather than the patient level, and observed patterns reflect short-term, behavioral responses rather than sustained adherence or clinical efficacy. The primary outcome focused on behavior changes within a 60 min window following notification delivery and does not capture longer-term adherence patterns, or downstream clinical outcomes such as wound healing. Third, the study did not collect patient-reported outcomes, including measures of satisfaction, usability, or perceived burden of the notification system. Such data are essential for evaluating acceptability [48,50] and are recommended by major clinical guidelines (e.g., ADA Standards of Care, EASD position statements) as core components of patient-centered digital health interventions. Future studies should incorporate validated instruments to assess these dimensions alongside behavioral and clinical endpoints. Fourth, illustrative patient case examples were included to contextualize variability in responsiveness but were not intended for comparative analysis or causal inference. Fifth, while odds ratios with 95% confidence intervals were reported for notification pathway comparisons, the effect size magnitudes should be interpreted cautiously, given the small sample and exploratory design. Finally, the absence of randomized comparison conditions limits conclusions regarding the relative contribution of notification intent versus underlying factors. Confounding factors and behavioral influences, including ulcer severity, comorbidities, socioeconomic factors, technology and digital literacy, and behavioral reactivity to monitoring (e.g., Hawthorne effect [51]), should be evaluated in future studies. Collectively, these limitations underscore that the findings should be interpreted as descriptive, hypothesis-generating signals of feasibility and behavioral responsiveness rather than evidence of causal or generalizable effects.

## 5. Conclusions

This study demonstrates the feasibility of implementing a mobile-based digital notification system integrated with real-time sensing technology to support offloading adherence in patients with active DFU. As a subgroup feasibility analysis of the larger SmartBoot parent study, this work was intended as a proof-of-concept to inform the design of future, larger-scale investigations. Despite the limited sample size, this study provides novel insights, as it is, to our knowledge, the first to evaluate a context-aware chatbot intervention in patients with active DFU during wound care over a longitudinal period of up to 12 weeks. The analysis of objective adherence responses to 130 digital notifications offers an initial framework for defining adherence dynamics and response patterns in this population. These findings provide proof of concept evidence for incorporating real-time behavioral signal detection and conversational AI into outpatient DFU management. While not designed to evaluate clinical outcomes or establish causal relationships, this early-phase work highlights the potential for monitoring and guiding patient engagement. Larger, adequately powered studies are needed to determine whether such interventions lead to sustained adherence and improved clinical outcomes before broader clinical generalization.

## Figures and Tables

**Table 1.** Baseline demographic, clinical, and wound characteristics of the study cohort. Participant demographics and comorbid conditions are summarized alongside wound characteristics, diabetes-related factors, functional status, and education.

Characteristic	Value
Demographics and Clinical History	
Age (years), mean ± SD	58 ± 6
BMI (kg/m^2^), mean ± SD	29.0 ± 5.2
Sex, n (%)	Male: 8 (100%)
Marital status, n (%)	Cohabitant: 6 (75%); Single: 2 (25%)
Ethnicity, n (%)	Hispanic: 8 (100%)
Race, n (%)	White: 8 (100%)
Hypertension, n (%)	6 (75%)
Falls in past year, n (%)	3 (37.5%)
Heart disease, n (%)	1 (12.5%)
Sleep problems, n (%)	1 (12.5%)
Stroke or cancer history	None
Wound Characteristics	
Wound age (days), mean ± SD	93 ± 117
Ulcer location, n (%)	
Midfoot	4 (50%)
Hindfoot	3 (37.5%)
Forefoot	1 (12.5%)
UT classification, n (%)	
Grade 1A	7 (87.5%)
Grade 2A	1 (12.5%)
Diabetes & Social Factors	
HbA1c (%), mean ± SD	8.5 ± 1.5
Diabetes duration (years), mean ± SD	16.9 ± 10.7
Functional independence, n (%)	
Independent	4 (50%)
Dependent	4 (50%)
Education level, n (%)	
High school or less	4 (50%)
College level and higher	4 (50%)

**Table 2.** Notification categories and corresponding message types used during the study. Schedule-based notifications were delivered at predefined times independent of real-time behavior, while context-aware notifications were triggered based on SmartBoot sensor-detected offloading behavior. Examples are provided to illustrate message intent and structure.

Notification Categories
Schedule-Based Notifications
Motivational Coaching (S1): Scheduled motivational messages at set intervals, independent of the patient’s activity state.
Example: Finish Strong Today. A few more hours in your SmartBoot this evening.
Safety and Technical Assurance (S2): System checks for technical connectivity and compliance (e.g., charging devices).
Example: To keep your ulcer protected, please put your SmartBoot on now. Make sure your watch and boot are charged. If you’re having trouble with comfort or device pairing, we can troubleshoot together.
Context-Aware Notifications
Clinical Course Correction (C1): Corrective prompts are triggered when the activity deviates from the prescribed protocol.
Example: Let’s Bring Back Your Streak! Remember how strong your routine was last week.
Adaptive Reinforcement (C2): Messages triggered by detected positive behavior or “near-goal” achievements.
Example: You did great this morning. Don’t Lose Your Progress! Can you put your SmartBoot back on?

**Table 3.** Sensor-defined behavioral response rates for digital health notifications stratified by delivery strategy (context-aware vs. schedule-based) and notification intent. Behavioral response is reported as % (n/N), where *n* is the number of notifications followed by a sensor-confirmed nonadherence-to-adherence transition within 60 min of delivery and *N* is the number of eligible notifications delivered during nonadherence at the time of delivery.

Notification Pathway	Notification Intent	Behavioral Response, % (n/N)

Schedule-based	Motivational Coaching (S1)	30.8% (12/39)
	Safety and Technical Assurance (S2)	40.7% (11/27)

Context-Aware	Clinical Course Correction (C1)	71.4% (20/28)
	Adaptive Reinforcement (C2)	77.4% (24/31)

## Data Availability

The raw data supporting the conclusions of this article will be made available by the authors on request.
